# Municipal Representation on Hospital Committees

**Published:** 1921-01-22

**Authors:** 


### Municipal Representation on Hospital Committees.

Hackney Borough Council has been in communication with the secretary of the City of London Maternity Hospital with regard to the Borough Council being represented upon the management committee of the hospital. The secretary says the hospital committee would be pleased to furnish letters of recommendation for use on behalf of necessitous cases in Hackney in respect to grants made by the Borough Council from time to time, but points out that the question of municipal representation upon the committee is a matter for the governors of the hospital, who elect the members of the committee at their annual court. The procedure necessary in order to put forward a nominee for election at the annual court would be for the Borough Council to nominate three persons (in respect of its recent grant of £39 18s.) to exercise the privilege of life governorship. These nominees would, in due course, receive notice of the holding of the annual court, and would then have the opportunity of attending and submitting the name of the desired representative of the Borough Council for election on the hospital committee of management. The Health Committee of the Borough Council reports that it considers that where money is contributed out of public funds a representative of the contributors should have the opportunity of attending meetings, not with a view to criticism but to become acquainted with the method of management. Further, the Health Committee considers that such attendance should have the effect of increasing public interest in the institution concerned and indirectly lead to the securing of further voluntary contributions. The committee, therefore, suggests that three lady members of the Council's Maternity Committee should be nominated for life governorship.

